# Survey of *Brucella* infection and malaria among Abattoir workers in Kampala and Mbarara Districts, Uganda

**DOI:** 10.1186/1471-2458-13-901

**Published:** 2013-09-30

**Authors:** Immaculate Nabukenya, Deogratius Kaddu-Mulindwa, George William Nasinyama

**Affiliations:** 1 Department of Biosecurity, Ecosystems and Veterinary Public Health, College of Veterinary Medicine, Animal Resources and Biosecurity, Makerere University, Kampala, Uganda; 2 Department of Medical Microbiology, School of Biomedical Sciences, College of Health Sciences, Makerere University, P.O. Box 7072, Kampala, Uganda; 3 Directorate of Research and Graduate Training, Makerere University, Kampala, Uganda

**Keywords:** *Brucella*, Malaria, Brucellosis, Abattoir workers, Zoonoses, MAT, STAT, Seropositivity

## Abstract

**Background:**

Brucellosis is among the most widespread zoonotic infections estimated at 14% in Uganda. A cross-sectional study was conducted to estimate the sero-prevalence, risk factors of *Brucella* infection and malaria among abattoir workers.

**Methods:**

A survey was conducted among 232 abattoir workers in main abattoirs of Kampala and Mbarara districts in February 2007. A pre-tested questionnaire captured socio-demographic and occupational data. Brachial vein blood was tested for *Brucella* using Microplate Agglutination Test (MAT) and Standard Tube Agglutination Test (STAT) with a cut off titre of 1:160, and giemsa stained blood slides for malaria. Data was analyzed in SPSS 17.0.

**Results:**

Seven males (3%, n = 232) had malaria and dual brucella and *Plasmodium falciparum* malaria was found in one person. *Brucella* sero-positivity was 10% (95% CI 6 – 16; n = 232) with 12% (n = 161) in Kampala and 7% (n = 71) in Mbarara district. Non-use of protective gear Odds ratio (OR 3.3, 95% CI (1.25 – 50) and working in the abattoir beyond 5 years OR 2.4 95% CI (1.4 – 5.6) were associated with increased risk of *Brucella* infection. Age, sex, religion, keeping animals and consumption of raw milk or products were not significant.

**Conclusions:**

*Brucella* infection is a real risk among abattoir workers and use of full protective gear reduced risk significantly. Sensitization and public health care programs are needed to control this emerging problem.

## Background

Brucellosis is among the most widespread zoonotic infections causing human suffering and economic losses in livestock [1-3]. However, it is often a neglected cause of morbidity in many regions of the world [3,4]. The disease is most common in rural areas among those involved in animal husbandry, meat-packers, dairy workers, veterinarians, consumers of unprocessed dairy products and in urban livestock keeping populations [2,5]. *Brucella* infection is transmitted from animals (cattle, goats, pigs, sheep, camels and buffaloes) to humans by bacteria belonging to the genus *Brucella. B. abortus*, *B. suis*, and *B. melitensis* are the causative agents, which affect cattle, swine, goats and sheep respectively are most pathogenic to humans [1]*. B. canis* and marine species also have zoonotic potential but are not as pathogenic.

The global burden of human brucellosis remains enormous with more than 500,000 infections per year worldwide [4-6]. Brucellosis has been reported in the Middle East [7], Mediterranean region [5], Northern and Sub-Saharan countries in Africa [4,6,8] with prevalence of 5-55% in humans and 8-46% in animals [9]. For example, in central Greece, prevalence was 32.49 cases/100,000 inhabitants [5]. In Iran, a Brucellosis endemic country, a study on brucellosis and HIV co-infection found a very high prevalence of 73% among HIV positive patients compared to 24% in HIV negative patients indicating a statistical difference in infection rates [7]. Among hospital patients in Markudi Nigeria, overall brucellosis prevalence was 7.6%, and 43.8% of these were abattoir workers and butchers [10]. Over 55% of 7161 people examined in different parts of Western Nigeria have positive *Brucella abortus* antibodies in their sera. Higher incidences of titres were found among dairy farmers and slaughter men than in the general population. The rates of infection among human and cattle populations in two farms studied were very similar [10]. In Egypt, incidence ranges from 0.28 to 70 per 100,000 population [4,11] and 11% prevalence among hospital patients [12].

Brucellosis is a highly prevalent disease in Uganda with 7 – 42.2% [2,13,14] among cattle and goats, posing a big threat to abattoir workers and consumers. A study done among hospital patients estimated 18-24% brucellosis prevalence [15] while it was estimated at 6 – 7% among herdsmen and consumers of raw milk and products [14]. In Kampala, Uganda, of 150 patients with joint pain, general malaise, and/or constant headache, 73% were found to be suffering from malaria and 13.3% from brucellosis showing a scenario often leading to misdiagnosis [16].

Few recent studies in Africa and globally have considered the abattoir workers as an occupational high risk group. A study among high risk groups in Erzurum, Turkey found higher infection among abattoir workers [1]. In India, assessment of 165 serum samples of abattoir associated personnel with dot-ELISA found 25.5%, 40% and 11% positive for brucellosis, listeriosis and tuberculosis respectively [17]. In Pakistan, Mukhtar and Kokab found 21.7% prevalence using ELISA with job category, age and duration in the abattoir as significant risk factors [18].

Diagnosis of brucellosis based on the clinical picture alone is difficult due to similarity with clinical presentations of other infections [3,9]. Symptoms and signs are non-specific and several other febrile illnesses, for example glandular fever, influenza, malaria and enteric infections may be simulated [11,18,19]. When an unusual complication is present, it may be overlooked [12]. Therefore, laboratory testing is an absolute prerequisite for proper diagnosis through blood culture and isolation of the causative organisms or serological testing [12]. However, culture requires special media, takes several weeks of incubation and has low sensitivity. Serological tests including the serum agglutination test (SAT or STAT), anti-human globulin test (Coombs test), complement fixation test (CFT) and Enzyme-linked Immunosorbent Assay (ELISA), therefore, are indispensable for an accurate diagnosis [9].

Malaria is endemic in Uganda with prevalence as high as 70% in patients with pyrexia of unknown origin [16]. Few studies have focused on brucellosis prevalence in Africa [4,6] and misdiagnosis with common conditions such as malaria and typhoid remains a challenge. In Uganda, brucellosis prevalence and the occupation link is unknown to health workers, and malaria, a common tropical disease which sometimes clinically mimics brucellosis has not been widely studied in apparently healthy populations [12,16]. There is no published data on prevalence of brucellosis in Uganda among abattoir workers which is a high risk group and the study therefore sought to address this gap. At the same time, this study assessed the prevalence of malaria in this apparently healthy population.

## Methods

### Study area

The study was carried out in Kampala City Council abattoir (KCCA) in Kampala district and Mbarara Municipal Council abattoir (MCA) in Mbarara district. KCCA handles a big slaughter load from many parts of the country. MCA is located in the cattle corridor, with high cattle production, thus representing significant risk to the abattoir workers.

### Study design, participant enrolment and laboratory processing

The survey carried out among 232 apparently healthy abattoir workers in January to February 2007 used quantitative data collection methods. The sample size was calculated based on 95% confidence level and prevalence of 18% [20]. Adult men and women who had worked for at least three months in the abattoirs and consented to participate were selected, with a proportion of 87% and 92% of the workers in KCCA and MCA respectively enrolled into the study.

To identify risk factors, socio-demographic variables like age, sex, education level and religion were measured. In addition, occupational factors such as use of protective gear, animal species handled and work done in the abattoir, duration of exposure; health-related factors (malaria, malaria-like symptoms and previous use of antibiotics) were studied as the independent variables using a questionnaire. Brachial vein blood was used to make a thick blood smear which was giemsa stained to assess for malaria parasitaemia. Serological screening by Microplate Agglutination test (MAT) and confirmation by Standard Tube Agglutination Test (STAT) [21] using *B. abortus* antigens from Veterinary Laboratory Agency UK, was done in Mulago Hospital Microbiology laboratory. Samples with any agglutination by MAT were further processed by STAT to quantify the amount of agglutination and a titer of 1:160 or greater indicated seropositivity.

### Statistical analysis

Data was entered in Epidata 3.0, cleaned, exported and analyzed in Statistical Package for Social Scientists, SPSS version 17.0 (Apache software, 2007). Descriptive statistics to summarize the data as well as odds ratios and the 95% confidence intervals were computed. For risk factor analysis, stepwise backward logistic regression was used to fit the best model after assessing and controlling for interaction and confounding. After bivariable analysis, factors with less than p < 0.2 (Table 1) were included in the multivariable analysis.

**Table 1:** **Significant characteristics with *Brucella *infection at bivariable analysis**

**Variable**		**Frequency (/n)**	**Unadjusted OR (95% CI)**	**p value**
Age	30 yrs or less	10/118	1	0.097
	Above 30 yrs	14/114	1.2 (0.9 – 1.6)	
Protective gear	Full gear	1/11	1	0.05
	Not full gear	23/221	2.5 (1.1 – 22.0)	
Duration in abattoir	4 yrs & less	4/53	1	0.12
	Above 5 yrs	20/179	1.2 (1.1 – 1.4)	
Keep animals	No	7/169	1	0.14
	Yes	17/63	1.3 (0.9 – 1.9)	
District with abattoir	Mbarara	5/71	1	0.13
	Kampala	19/161	1.2 (1.0 – 1.4)	

### Ethical issues

Ethical approval to conduct the study was received from Makerere University Clinical Epidemiology Unit, Faculty of Medicine Research and Ethics Committee, administrators of KCCA and Mbarara Municipal Council abattoirs. Written consent was obtained from the participants. Participants with *Brucella* infection were referred to appropriate health facilities for treatment.

## Results

A total of 232 people who work in Kampala City Council abattoir, KCCA (161) and Mbarara Municipal Council abattoir, MCA (71) abattoirs were interviewed and tested for *Brucella* infection and malaria.

### Socio-demographics

The majority of the workers were males (78%, n = 232) and overall mean age was 32.7 +/-9 years (range 19–70 and median 30 years). Up to 91% of the workers had some education and 48% had at least attained secondary school education. The religion to which majority of the participants were affiliated was Islam (50%, n = 232) and 69% were married (Table 2). By occupation category in the abattoir, slaughterers of cattle, goats and sheep were 35%, followed by 19% who prepare food, meat traders (15%) and transporters (11%).

**Table 2:** **Seroprevalence of *Brucella *infection among abattoir workers in Kampala and Mbarara districts in different strata (n = 232)**

**Variable**	**(n = 232, %)**	**+ve Kampala (161, %)**	**+ve Mbarara (71, %)**
**Overall**	24 (10)	19 (12)	5 (7)
**Sex** Male	18 (8)	13 (8)	5(7)
Female	6 (3)	6 (4)	0 (0)
**Age** 20 and below	1 (1)	1 (1)	0 (0)
21 – 30	6 (4)	6 (4)	3 (3)
31 – 40	8 (5)	8 (5)	0 (0)
41 – 50	3 (2)	3 (2)	2 (3)
Above 50	1 (1)	1 (1)	0 (0)
**Education** None	3 (1)	2 (1)	1 (1)
Primary	7 (3)	5 (3)	2 (3)
Secondary	13 (6)	12 (8)	1 (1)
Tertiary	1 (1)	0 (0)	1 (1)
**Religion** Catholic	7 (3)	5 (3)	2 (3)
Protestant	4 (2)	3 (2)	1 (1)
Muslim	13 (6)	11 (7)	2 (3)
Born again	0 (0)	0 (0)	0 (0)
SDA	0 (0)	0 (0)	0 (0)
**Marital** Single	6 (3)	4 (3)	2 (3)
**status** Married	17 (7)	14 (9)	3 (4)
Separated	1 (1)	1 (1)	0 (0)
Divorced		0 (0)	0 (0)
**Keep** No	8 (3)	2 (1)	4 (6)
**animals** Yes	16 (7)	17 (11)	1 (1)
**Malaria** No	23 (10)	2 (1)	0 (0)
**in past 3** Yes	1(1)	17 (7)	5 (7)
**Months**			
**Raw milk** No	8 (3)	6 (4)	2 (3)
**/products** Yes	16 (7)	13 (8)	3 (4)
No	21 (9)	18 (11)	5 (7)
**Protective gear** Yes	3 (2)	1 (1)	0 (0)
**Brucellosis** No	6 (3)	3 (2)	3 (4)
**(past 2 years)** Yes	18 (8)	16 (10)	2 (3)

Only 7 people (3%, n = 232), all males aged 25 – 35 years had *Plasmodium falciparum* malaria. In addition, there was a dual occurrence of *Brucella* infection and malaria due to *Plasmodium falciparum* in one person. The overall prevalence of *Brucella* infection was 10% (95% CI 6 – 16; n = 232). On gender desegregation, the prevalence of *Brucella* infection in females was 12% (n = 52) and 10% (n = 180) in males. The proportion of participants with *Brucella* infection among participants with at least secondary education and Muslims was each 13%. Kampala district had more *Brucella* seropositive abattoir workers (12%, n = 161) compared to Mbarara district (7%, n = 71), though not significantly different (p = 0.08). Ninety percent (n = 24) of the workers who reported positive diagnosis and treatment for brucellosis in the past two years were still positive. The prevalence established in workers who consumed raw milk or products and those who do not was similar (10% and 11% respectively). A summary of the results is in Table 2.

### Knowledge and past experience with brucellosis

The majority of the participants, 61% (n = 232) reportedly had heard of brucellosis and of these only 30% (n = 142) said they knew how the disease was transmitted. The most commonly cited modes of brucellosis transmission were ingestion of meat that is not well prepared and drinking raw milk (55%, n = 42 and 38% respectively). Past experience with brucellosis was reported by 11% (n = 232) while 23% (n = 232) reported that they knew someone who had suffered from brucellosis (Table 2). Undulant fever and joint pains were the most common symptoms reported 67% and 60% respectively (n = 67) as shown in Table 3.

**Table 3:** Reported symptoms of brucellosis previously experienced by abattoir workers or patients they knew had suffered from brucellosis (n = 67)

**Symptom**	**Frequency**	**Percentage**
Undulant fever	45	67
Joint pains	40	60
Back pain	31	46
Headaches	23	34
Weakness	23	34
Fatigue	14	21
Abdominal pain	11	16
Chills	11	16
Excessive sweating	9	13
Loss of appetite	8	12
Night sweats	7	10

### Risk factors

The majority of the participants reported using some protective gear when working (92%, n = 232) but only 5% (n = 214) had full protective gear. Seroprevalence of *Brucella* infection was up to 11% (n = 214) among abattoir workers without full protective gear. When asked how often they used the protective gear, 95%, (n = 214) reported routine use, twice or more times a week by 4% and once a week by 0.9%.

Only 35% (n = 232) of the participants consumed raw milk or its products, with 7% brucellosis seropositivity in this proportion. Of the 81 participants who drank raw milk and/or unprocessed products, 41% drank raw milk while 17% ate uncooked cow ghee. Results showed that only 27% (n = 232) kept animals at home, of whom 28% (n = 61) kept goats; 25% had cattle; 15% kept dogs and cats. Interaction of the workers with these animals was during grazing or feeding.

Use of full protective gear; odds ratio, OR 0.3 (95% CI 0.02 – 0.8) and duration of more than five years in the abattoir, OR 2.4 (95% CI 1.4 – 5.6) were associated with reduced and high risk for infection respectively. The final model predicting *Brucella* seropositivity is presented in Table 4.

**Table 4:** **Final model predicting *Brucella *seropositivity among abattoir workers**

**Variable**	**Coefficient**	**OR (95% CI)**	**p-value**
Keep animals	2.4	1.1 (0.3 – 4.2)	0.190
No protective gear	1.26	3.3 (1.25 – 50)	0.02**
District	0.22	2.1 (0.8 – 5.4)	0.135
Duration of exposure	1.54	2.4 (1.4 – 5.6)	0.04**
Age	2.60	1.1 (0.03 – 10.4)	0.13
Constant	8.3		0.105

**Significant predictors of *Brucella* seropositivity at p < 0.05.

## Discussion

The low prevalence of malaria (3%) is possibly because this was a healthy population and the blood used was from the brachial vein rather than the finger tip at the periphery where more parasites sequestrate. Information on whether the malaria positives workers had taken malaria pills over the last few days prior to commencement of this study was not collected. This would provide a plausible explanation to the low prevalence. However, Maichomo and others reported 9% prevalence of malaria and 13% brucellosis in 488 patients with flu-like illnesses and higher prevalence is reported in patients rather than healthy populations [22]. The overall seroprevalence of *Brucella* infection in this study was high, with one in every ten abattoir workers seropositive. This prevalence parallels that in animals (8 – 46%) which are the primary source for the abattoir workers [2]. Similar studies among abattoir workers elsewhere have found similar results [1,18,23]. Dual occurrence of malaria and brucellosis has been reported before in a traveler from Chad to Europe [19]. There were similar levels of exposure among abattoir workers and herdsmen who deal with animals from different areas of brucellosis endemicity.

Since brucellosis indirectly causes infertility through abortion, this is a reason for sale of such animals. In addition, 10% (24) of the participants attested to having been diagnosed and treated for brucellosis before. Since the study was a serosurvey, the tests used did not discriminate between current active and past infections. The reactive proportion therefore was still part of the overall prevalence obtained. What is strange is that despite the high prevalence of *Brucella* infection in humans, it is not considered for routine laboratory referrals in cases of acute febrile illness. The low prevalence of malaria may indicate the need to consider brucellosis in the high risk populations presenting with febrile illnesses.

The proportion of individuals who had full protective gear (gloves, white coat/overall, gumboots and or no head gear) and were *Brucella* seropositive was small (9%) compared to that without (23%). Also those without full protective gear were about three times as likely to be *Brucella* seropositive as those with full protective gear. However, 9% seroprevalence among those with full gear may be explained by inhalation as the mode of transmission [23].

Socio-demographic factors (age, sex, education level, religion) were all not significantly associated with *Brucella* infection unlike in other studies [1]. Although significantly higher prevalence was noted in males than females in a brucellosis and HIV co-infection study [7], another study found no association between brucellosis and sex or age although females were more affected than males and those with age in the second and fourth deciles were more affected [10]. This latter and our findings do not concur with other studies which found age and gender associated significantly [8,24]. Other factors like duration of exposure and type of work done in the abattoir were also not statistically significant, although 57% had worked in the abattoir for six to 15 years.

This study being a cross sectional one had no temporal background and was not able to establish the causal relationship between brucellosis and possible factors associated. Owing to the fact that assessment of the factors was through self report by the participants responses to a questionnaire, there was a potential bias due to underreporting. Measurement bias was reduced by the two tests which improved on the specificity and included less false positives. No factor was found to cause interaction or confounding.

## Conclusion

The seroprevalence of *Brucella* infection is high, suggesting that one in ten abattoir workers is infected. In order to reduce *Brucella* infection, abattoir workers need to use full protective gear. Sensitization of abattoir workers, management and the general population about brucellosis will help in effective control and prevention.

## Abbreviations

KCC: Kampala City Council; STAT: Standard tube agglutination test; MCA: Mbarara Municipal Council Abattoir; MAT: Microplate agglutination test.

## Competing interests

The authors declare that they have no competing interests.

## Authors’ contributions

NI, KMD and NGW were involved in design of the study as well as in manuscript write up. KMD and NGW supervised the field and laboratory work. NI participated in data analysis and write up. KMD is a microbiologist, majorly bacteriology with a veterinary background. As a Professor, he has supervised many students with several research grants in this area. Currently focusing on Enterobacteriaceae, he continues to inspire many through research, teaching and grant management. NGW has conducted a lot of research in the area of zoonoses including brucellosis and tuberculosis in urban and peri-urban settings of Uganda. He has supported many students and junior researchers in areas of epidemiology, public health and preventive veterinary medicine. All authors read and approved the final manuscript.

## Pre-publication history

The pre-publication history for this paper can be accessed here:

http://www.biomedcentral.com/1471-2458/13/901/prepub
